# Case control study of the geographic variability of exposure to disinfectant byproducts and risk for rectal cancer

**DOI:** 10.1186/1476-072X-6-18

**Published:** 2007-05-29

**Authors:** Gerald E Bove, Peter A Rogerson, John E Vena

**Affiliations:** 1 Department of Geography, University at Buffalo, Wilkeson Hall, Buffalo, NY 14261, USA; 2 Department of Biostatistics, University at Buffalo, Farber Hall, Buffalo, NY 14261, USA; 3 Department of Epidemiology and Biostatistics, Arnold School of Public Health, University of South Carolina, 800 Sumter Street, Columbia, SC 29208, USA

## Abstract

**Background:**

Levels of byproducts that result from the disinfection of drinking water vary within a water distribution system. This prompted us to question whether the risk for rectal cancer also varies, depending upon one's long term geographic location within the system. Such a geographic distribution in rectal cancer risk would follow naturally from an association between level of byproduct and rectal cancer risk. We assess the effects of estimated geographic variability in exposure to some of the components of the trihalomethane group of disinfectant byproducts (DBPs) on the odds ratios and probabilities for rectal cancer in white males in a case control study of 128 cases and 253 controls, conducted in Monroe County, Western New York State, U.S.A. The study was designed around health data initially collected at the University at Buffalo (Department of Social and Preventative Medicine) as part of the Upstate New York Diet Study, and trihalomethane (THM) data collected from a separate independent study of THMs conducted by Monroe County Department of Health. Case participants were chosen from hospital pathology records. The controls are disease-free white males between 35–90 years old, living in Monroe County, and chosen from control groups for studies from cancer of five other (unrelated) sites. Using a combination of case control methodology and spatial analysis, the spatial patterns of THMs and individual measures of tap water consumption provide estimates of the effects of ingestion of specific amounts of some DBPs on rectal cancer risk. Trihalomethane (THM) data were used to spatially interpolate levels at the taps of cases and controls, and odds ratios were estimated using logistic regression to assess the effects of estimated THM exposure dose on cancer risk, adjusting for alcohol, dietary beta carotene intake, tap water intake, and total caloric intake.

**Results:**

Trihalomethane levels varied spatially within the county; although risk for rectal cancer did not increase with total level of trihalomethanes, increasing levels of the component bromoform (measured in ug/day) did correspond with an increase in odds ratios (OR = 1.85; 95% CI = 1.25 – 2.74) for rectal cancer. The highest quartiles of estimated consumption of bromoform (1.69–15.43 ug/day) led to increased risk for rectal cancer (OR = 2.32; 95% CI = 1.22–4.39). Two other THMs were marginally associated with an increase in risk – chlorodibromomethane (OR = 1.78, 95% CI = 1.00–3.19) and bromodichloromethane (OR = 1.15; 95% CI = 1.00–1.32).

**Conclusion:**

Levels of THMs in the water distribution system exhibited spatial variation that was partially due to variation in water age. We also observed a geographic pattern of increased risk of rectal cancer in areas with the highest levels of bromoform in the county.

## Background

At a global scale there are geographic differences in the prevalence of rectal cancer, and the highest rates generally occur in economically developed areas (e.g., Australia, Japan, New Zealand, and North America) compared with less developed areas (e.g., Africa and China). This is most often explained by environmental factors related to diet [1]. There are also geographic disparities within the United States; for example, Devesa et al. [2] summarized geographic patterns of urinary bladder and rectal cancers in the U.S. for the period 1950–94 and noted that throughout the period, high rates clustered in the northeastern United States. Other potential risk factors for rectal cancer include tobacco consumption [3,4], alcohol consumption [5,6], genetic disposition [7,8], occupational exposures [9,10], diet [5,11-13], and disinfectant by-products (DBPs), the focus of the present study.

DBPs in water supplies are formed from the interaction of organic material in raw water and an introduced disinfectant. DBPs are particularly problematic in surface water supplies since they generally contain the largest amount of organic material [14]. Early work, published soon after their discovery, found that some of the byproducts of the disinfection process are carcinogenic [15-18]; however, the carcinogenic potential of many DBPs remains unknown [19,20]. The geographic component of risk stems from the well-established association between DBP formation and pipe retention time [21-24]. If other post-disinfection variables are held constant (pipe condition, temperature etc.), DBP levels generally increase with increasing post-disinfection time (often related strongly to distance), measured from the fresh water treatment plant. Although the relationship between retention/reaction times and DBP formation is a complex one with many possible covariates, in an open pressure water distribution system (one in which greater distance from the treatment plant implies longer pipe retention times) we would expect the highest levels of DBPs to be at those areas at the far reaches of the network. This possible space-time relationship prompted us to examine if there were geographic disparities in the probability of developing rectal cancer within a single distribution system. Although much of the focus concerning the carcinogenic potential of DBPs has centered on the urinary bladder [25-27], some studies have demonstrated a relationship between DBPs and cancers of the colon and/or rectum [28-31], kidney [32], and possible adverse effects on birth outcomes [33-35].

Although mouse models generally support carcinogenicity of some THMs in the intestine, results of studies in human populations are mixed. Evidence in support of carcinogenicity of exposure to disinfected water in the course of everyday activities in human populations [27,29,36-40] is offset by equally compelling studies [30,41,42] suggesting no significant relationship. In an analysis of six rectal cancer studies conducted prior to 1992, Morris [43] reported a pooled risk of 1.38 for exposure to DBPs. In an earlier study conducted in the county adjacent to where the present study was conducted, Carlo [44] found no association between areas of increased THM levels and rectal cancer. Most similar to the present study, King and Marrett [30] accounted for individual THM intake and found that risk increased for colon cancer as years of exposure to THM increased, but found no such relationship for rectal cancer. Results of the present study suggest that lack of consistent statistical evidence of rectal cancer risk may be at least partially the result of not differentiating exposure to various components of the THM mixture. Rodent studies [45,46] suggest the brominated compounds to be most carcinogenic in the intestine; however, the brominated compounds are only a component (generally a less volatile component than many other THM compounds, perhaps making it more likely to be consumed in drinking water vs. other routes of exposure) of the total trihalomethanes often used to test exposure-based risk in humans. A 1985 study [47] indicates assessment of relevant individual exposure to organics in drinking water a "thorny barrier to progress" in assessing the risk associated with development of cancer in the digestive tract, and this issue continues to be a challenge to assessing DBPs and cancer risk. We have attempted in the present study to provide some refinement of exposure measurement from previous studies of drinking water and cancer by differentiating and assessing some individual trihalomethane compounds; however, case sample size is limited, and assumptions have been made that also reduce the power of the present study as well.

## Methods

### Study design and subjects

The study was designed around case control data initially collected at the University at Buffalo (Department of Social and Preventative Medicine) as part of the Upstate New York Diet Study. Trihalomethane data for our study were supplied by Monroe County Water Authority (MCWA) and the Monroe County Health Department, and were generated from work unrelated to the Diet Study. For the present study, all 128 case participants (originally chosen from hospital pathology records) from Monroe County were selected as a subset from the three-county Diet Study. We confined the study to this area because of the availability of THM data. Controls (253) are disease-free white males between 35–90 years old, living in Monroe County, and chosen from control groups for studies from cancer of five other sites: oral cavity, esophagus, stomach, larynx, and lung. We were interested in evaluating the effects of residential address on the levels of trihalomethanes (THMs) at the tap, total daily tap water consumption, and the specific amount of additive exposure to trihalomethanes given by the effect of these two variables of interest on the odds ratio that study participants were cases or controls. Because we used a geographically-based exposure variable (THM levels), we chose not to include any address-matched controls from the initial rectal cancer study [5]. More detail on the Upstate New York Diet Study, including case and control selection, and dietary assessment methodology is given elsewhere [5,48-50]. Distributions of covariates for cases and controls were similar (Table 1).

**Table 1:** Background information (mean, standard deviation) for cases and controls

	**Cases (128)**	**Controls (253)**
Age(yrs)	64.3(± 9.0)	64.4(± 9.6)
Education (yrs)	12.1(± 3.0)	12.4(± 3.3)
Height(cm)	172.7(± 7.1)	173.7(± 7.3)
Weight(kg)	79.1(± 13.2)	79.2(± 12.1)
Daily tap water drinking volume (*l*)	1.9(± 0.9)	1.8(± 0.9)
Total yrs. Public water	56.3(± 17.3)	60.2(± 15.3)
% life at present water source	88(± 1.8)	91(± 1.1)

The Upstate Diet Study sought to assess the association between several cancer types and the following dietary factors: vitamins, dietary fat, fiber, protein, caloric intake, and commonly consumed non-nutritive items such as coffee and tea. Covariate data for both cases and controls were collected via nurse interviewers during extensive 2.5 hour interviews conducted from April 1978 – April 1986 [5]. Dietary and other data (including current address and occupation, residential and occupational history, water source (current and historical), and health history) were taken with a modified food frequency questionnaire, including intake on 129 foods, alcohol, and all other fluid consumption, marital status, and other background data not pertinent to the current study. From these data, indices of energy, fiber, and 15 nutrients were calculated according to published food guidelines [5]. Survey instruments were identical for all cases and controls. Cases were asked about their dietary habits one year prior to diagnosis and controls were asked about their usual dietary habits for the year prior to interview.

### Study area and drinking water treatment and distribution

The geographic study area is Monroe County, located in Western New York State (USA) on the shore of Lake Ontario. Population of the county is approximately 753,000, including one major city (Rochester, pop. 219,000). Both cases and controls within Monroe County receive water from Hemlock Lake and Lake Ontario via the City of Rochester water treatment and distribution system, and/or the Monroe County Water Authority treatment and distribution system, with MCWA serving the majority of the population. Trihalomethane data provided by the Monroe County Health Department (MCHD) were used in conjunction with case control data to determine THM exposure. MCWA distributes about 60 million gallons of water per day to 163,000 retail and 650,000 wholesale customers. MCWA treatment system uses granular activated carbon as a post-chlorination filter and Cl^+2 ^as a disinfectant (known to produce byproduct THMs, haloacids, and haloacetonitriles [23]). Distribution takes place within 2500 miles of pipes, with system retention times through this network ranging from 5–30 days or more, and average times of 5–7 days (source: personal communication with Dick Metzger, engineer at MCWA).

### THM data

MCHD initiated a study of trihalomethanes in the water treatment and distribution systems in the county in 1998. MCHD tested public water supplies seven to ten or more times per year (through all four seasons) at approximately 65 sample sites within the county for the THMs bromoform, bromodichloromethane, chloroform, chlorodibromomethane. In addition, a measurement is made for "Total 551", which is a composite measure that includes 12 DBPs, based upon EPA's method 551 for testing organic compounds in drinking water [51]. Mean and median values for all THM samples are given in Table 2. Testing for Total 551 began in 1998 and continued until 2003; other THMs were sampled over a two or three-year period from 2000 to 2003. Approximately 80% of the sample locations for the THM study were located within the MCWA area of service; the remainder were within the City of Rochester water system. Exchange of water between these two systems can occur during peak demand times and often involves bulk water purchased from MCWA and distributed by Rochester Water Authority.

**Table 2:** Mean and median of sampled THM levels (ug/*l*)

	**Bromoform**	**Bromodichloro-methane**	**Chloroform**	**Chlorodibromo-methane**	**Total 551**
Mean	1.30	8.72	19.75	4.11	35.07
Median	0.45	8.48	17.61	3.80	33.00

### GIS THM modelling

Addresses of THM sample collection sites were provided by MCHD and were used together with residential addresses from the case control study using GIS. Case control addresses allowed for determination of the location of each study participant in the water distribution system in relation to the water sample points.

**Figure 1:**
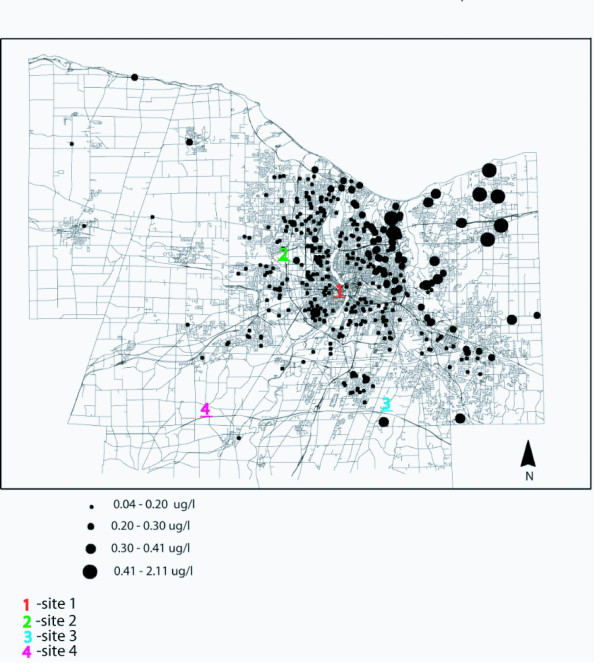
**Bromoform level estimates from kriging model, and locations of long term sample sites indicated in Figure 4. **Note that levels represent average annual values. The highest level measured for bromoform within a single sampling period was > 10.0 ug/l.

**Figure 2:**
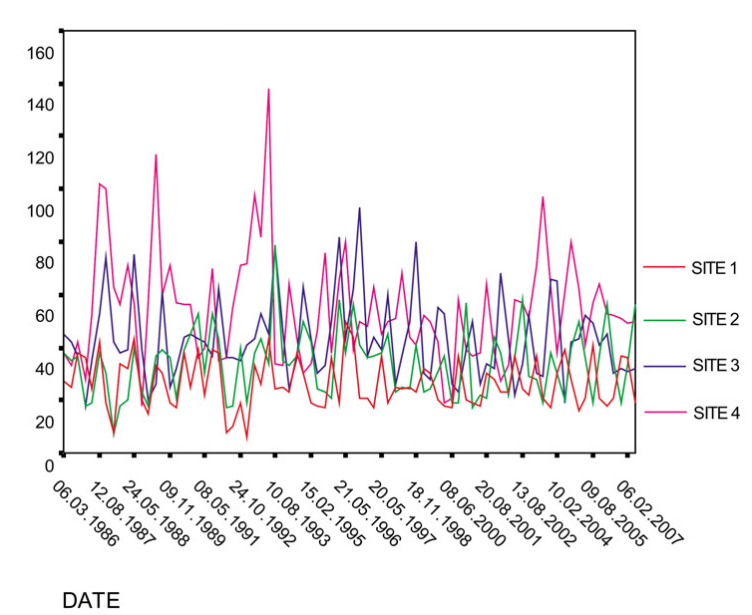
Historic (1986 – 2007) total trihalomethane levels at four sample sites in Monroe County, sample sites shown in Figure 1.

The THM study included a wide range of measured values at each sample location that reflected seasonal variation. We obtained data for four sample locations within the system that have been sampled since 1986 (approximately a twenty year period). These sample locations are indicated in Figure 1 and correspond to the graph in Figure 2. Although the graph of long term measured values for total trihalomethanes (Figure 2) indicates substantial seasonal variation, long term averages have not experienced substantial change.

From MCHD's THM data we calculated the mean value for each sample point. The resulting values were then used in kriging, a method of interpolation, to provide a surface of THM values from which we were able to extract estimates for THM levels at the locations of cases and controls in the study. These were then used in conjunction with data from the case control study to determine rectal cancer risk from Total 551, and from each THM component for which estimates were available. There was a strong geographic pattern of THM levels (Moran's *I *= 0.04–0.82) that was linked to water age and distance from treatment source [52]. We found a highly significant (*p *< 0.001) Pearson correlation coefficient (.251) between water age at sample sites and THM levels. Although the magnitude of the coefficient is small, the large sample size allows rejection of the null hypothesis that water age and THM levels are unrelated.

### Statistical analysis

We used an unconditional maximum likelihood logistic model formulated using a hierarchical backward elimination approach. Choice of covariate inclusion and model construction proceeded mindful of [5], a study that focused on the relationship between dietary factors and rectal cancer.

We tested a host of possible covariates in numerous combinations. These included sodium intake, education, age, ethnic background, occupation, weight, BMI, dietary fiber intake, present weight, weight (3,2, and 1 year) prior to interview, total caloric intake, calories from fat, retinol intake, tap water intake, age, education, water intake from foods, pack years of cigarette smoking and other forms of tobacco consumption, total monthly alcohol consumption (all types, including beer, wine and hard liquor), and beta carotene intake as determined by [5]. In our results, which we present below, we have adjusted for consumption of alcohol, beta carotene, and total calories. The other covariates were either not significant or had incorrect signs, and were therefore omitted from the model. For example, fiber and beta carotene are highly correlated in the data, leading to an incorrect sign for the fiber variable; hence there was no need to include both in the final set of logistic regression models.

In assessing the potential effects of THMs, we include in each model the following three variables: (a) an estimate of THMs at the location of the case or control, (b) an estimate of tap water consumption, and (c) an interaction variable based upon the product of the previous two; that is, a composite exposure variable representing consumption of THMs per day.

Quartiles were created for bromoform exposure based on the total daily consumption of bromform calculated as the product of total daily tap water consumption in liters and the bromoform level (ug/l) estimates determined from the kriging model. Beverages included in the total tap water consumption variable included coffee, tea (hot and iced), 75% of reconstituted frozen orange juice, all other juices (made with tap water), and glasses of water taken directly from the tap. Dietary sources of tap water were divided further into beverages usually made with boiled tap water, such as coffee and tea, and beverages made with unheated tap water. Triahlomethane data provided by the Monroe County Health Department were given in ug/l, and consumption data were expressed as cups per day. To determine total ug/day trihalomethane consumption, total daily tap water consumption data (cups) were converted to liters, based on 8 ounces of fluid per cup, and 33.814 ounces per liter. These consumption data were then multiplied by THM estimates to determine total consumption of ug of individual THMs per day.

### Model formulation for estimation of odds ratios for individuals

**Figure 3:**
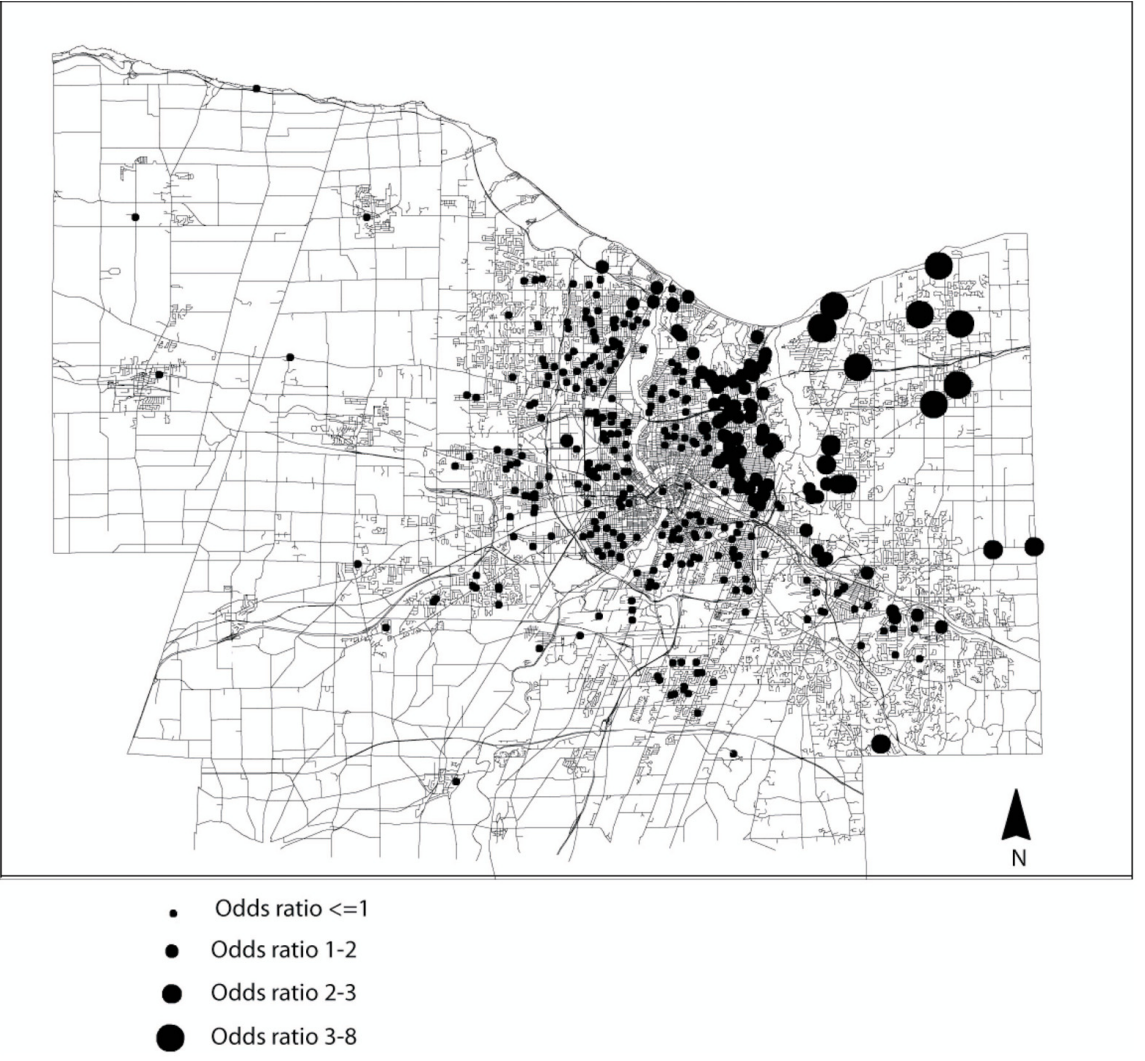
**Individual odds ratios for rectal cancer risk for exposure to the THM bromoform**. Note: Dependent variable determined as total daily ingestion of bromoform (ug/l) given as daily tap water intake (ug/l) and total bromoform contents of tap water (ug/l). Adjusted via assigning "average" values for covariates

To determine odds ratio estimates for "average" individuals residing at each location in the study (Figure 3), mean values for covariates (monthly alcohol intake in ounces, beta carotene intake, total caloric intake, and fiber) were used in the following expression for the odds, together with the location-specific THM estimate:

eβ0+β1x1+...+βnxn+βTHMTHM
 MathType@MTEF@5@5@+=feaafiart1ev1aqatCvAUfKttLearuWrP9MDH5MBPbIqV92AaeXatLxBI9gBaebbnrfifHhDYfgasaacH8akY=wiFfYdH8Gipec8Eeeu0xXdbba9frFj0=OqFfea0dXdd9vqai=hGuQ8kuc9pgc9s8qqaq=dirpe0xb9q8qiLsFr0=vr0=vr0dc8meaabaqaciaacaGaaeqabaqabeGadaaakeaacqWGLbqzdaahaaWcbeqaaGGaciab=j7aInaaBaaameaacqaIWaamaeqaaSGaey4kaSIae8NSdi2aaSbaaWqaaiabigdaXaqabaWccqWG4baEdaWgaaadbaGaeGymaedabeaaliabgUcaRiabc6caUiabc6caUiabc6caUiabgUcaRiab=j7aInaaBaaameaacqWGUbGBaeqaaSGaemiEaG3aaSbaaWqaaiabd6gaUbqabaWccqGHRaWkcqWFYoGydaWgaaadbaGaemivaqLaemisaGKaemyta0eabeaaliabdsfaujabdIeaijabd2eanbaaaaa@4B91@

This yields an estimate of the odds that, at a specific location, an individual with "average" characteristics on the covariates, and exposure to the mean THM level at that location, is a case. Given that the ratio of cases to controls as 128/253, it is approximately twice as likely that choosing randomly from the sample set would yield a control vs. a case. Therefore to phrase the odds of being a case in terms of the risk of rectal cancer, we divided the resulting odds by 128/253. The resulting odds are mapped in Figure 3.

## Results

Cases and controls were nearly identical in age, education, height, weight, and tap water consumption (Table 1). Controls had a slightly longer history using public water sources than cases. Both cases (88%) and controls (91%) spent the majority of their life consuming water at the source indicated during sampling.

We first examined the effect of a measure of total THMs (Total 551) on increased risk of rectal cancer. The results in Table 3 show that, after adjusting for covariates, there is no apparent significant effect of tap water consumption, THM level, or THM intake

**Table 3:** The effects of THMs (Total 551) and covariates on risk of rectal cancer

**Variable**	**OR**	**95% Confidence Interval**	***p***
Alcohol (1000 oz./month)	1.64	(1.09 – 2.47)	0.018
Betacarotene (10,000 IU/month)	0.58	(0.43 – 0.78)	<0.001
Total Calories (10,000/month)	1.18	(1.02–1.36)	0.022
Tap water (liters)	1.05	(0.67–1.84)	0.831
Total 551 estimate (ug/l)	1.01	(0.98–1.03)	0.518
Total 551 consumption (ug/day)	1.01	(0.99–1.03)	0.330

(eβ0+(β1X¯1)+...+(βn...X¯n...)+(βTHMTHM))
 MathType@MTEF@5@5@+=feaafiart1ev1aaatCvAUfKttLearuWrP9MDH5MBPbIqV92AaeXatLxBI9gBaebbnrfifHhDYfgasaacH8akY=wiFfYdH8Gipec8Eeeu0xXdbba9frFj0=OqFfea0dXdd9vqai=hGuQ8kuc9pgc9s8qqaq=dirpe0xb9q8qiLsFr0=vr0=vr0dc8meaabaqaciaacaGaaeqabaqabeGadaaakeaadaqadaqaaiabdwgaLnaaCaaaleqabaacciGae8NSdi2aaSbaaWqaaiabicdaWaqabaWccqGHRaWkcqGGOaakcqWFYoGydaWgaaadbaGaeGymaedabeaaliqbdIfayzaaraWaaSbaaWqaaiabigdaXaqabaWccqGGPaqkcqGHRaWkcqGGUaGlcqGGUaGlcqGGUaGlcqGHRaWkcqGGOaakcqWFYoGydaWgaaadbaGaemOBa4MaeiOla4IaeiOla4IaeiOla4cabeaaliqbdIfayzaaraWaaSbaaWqaaiabd6gaUjabc6caUiabc6caUiabc6caUaqabaWccqGGPaqkcqGHRaWkcqGGOaakcqWFYoGydaWgaaadbaGaemivaqLaemisaGKaemyta0eabeaaliabdsfaujabdIeaijabd2eanjabcMcaPaaaaOGaayjkaiaawMcaaaaa@5741@

per day on the risk of rectal cancer. Note that increasing consumption of alcohol and total calories increase risk, while increased consumption of beta carotene decreases risk. Tap water consumption by itself did not affect rectal cancer risk.

We next examined individual THM components, to assess whether they had effects on risk (Table 4). After adjusting for covariates, both bromoform level at the location of the case or control (OR 1.20; 95% CI 1.05–1.37), and bromoform consumption (OR 1.85; 95% CI 1.25–2.74) were significantly associated with increased risk for rectal cancer (Table 4). Not only do high levels of bromoform at the residential location lead to higher risk of rectal cancer, but this effect is enhanced among those who drink relatively higher amounts of tap water. Chlorodibromomethane was also marginally significant (OR = 1.78, 95% CI = 1.00–3.19), as was bromodichlromethane (OR = 1.15; 95% CI = 1.00–1.32); these results are also shown in Table 4. Chloroform was not significantly associated with increased risk for rectal cancer.

**Table 4:** The effects of bromoform, chlorodibromethane, and bromodichloromethane on risk of rectal cancer.

	**Bromoform**			**Chlorodibromo-methane**			**Bromodichloro-methane**			**Chloroform**		
	**OR**	**95% CI**	***p***	**OR**	**95% CI**	***p***	**OR**	**95%CI**	***p***	**OR**	**95%CI**	***p***
Alcohol (1000 oz./month)	1.77	(1.16 – 2.70)	0.008	1.65	(1.10–2.47)	.005	1.68	(1.12–2.52)	0.012	1.63	(1.09 – 2.44)	0.018
Betacarotene (10,000 IU/month)	0.56	(0.41–0.76)	<0.001	0.65	(0.50–0.84)	0.001	0.64	(0.50–0.84)	0.001	0.64	(0.49–0.83)	<0.001
Total Calories (10,000/month)	1.22	(1.05–1.40)	0.005	1.27	(1.14–1.42)	<0.001	1.26	(1.13–1.42)	<0.001	1.27	(1.14–1.42)	<0.001
Tap water (liters)	0.94	(0.73–1.20)	0.601	1.07	(0.45–2.59)	0.880	0.99	(0.83–1.16)	0.900	1.05	(0.52–2.12)	0.887
THM estimate (ug/l)	1.20	(1.05–1.35)	0.007	1.16	(0.85–1.58)	0.173	0.93	0.82–1.13)	0.465	1.00	(0.93–1.09)	0.777
THM consumption (ug/day)	1.85	(1.25–2.74)	0.002	1.78	(1.00–3.19)	0.052	1.15	(1.00–1.32)	0.047	1.00	(0.98–1.02)	0.908

"THM estimate" and "THM consumption" refer to the effects of individual THM components specified in each column.

**Figure 4:**
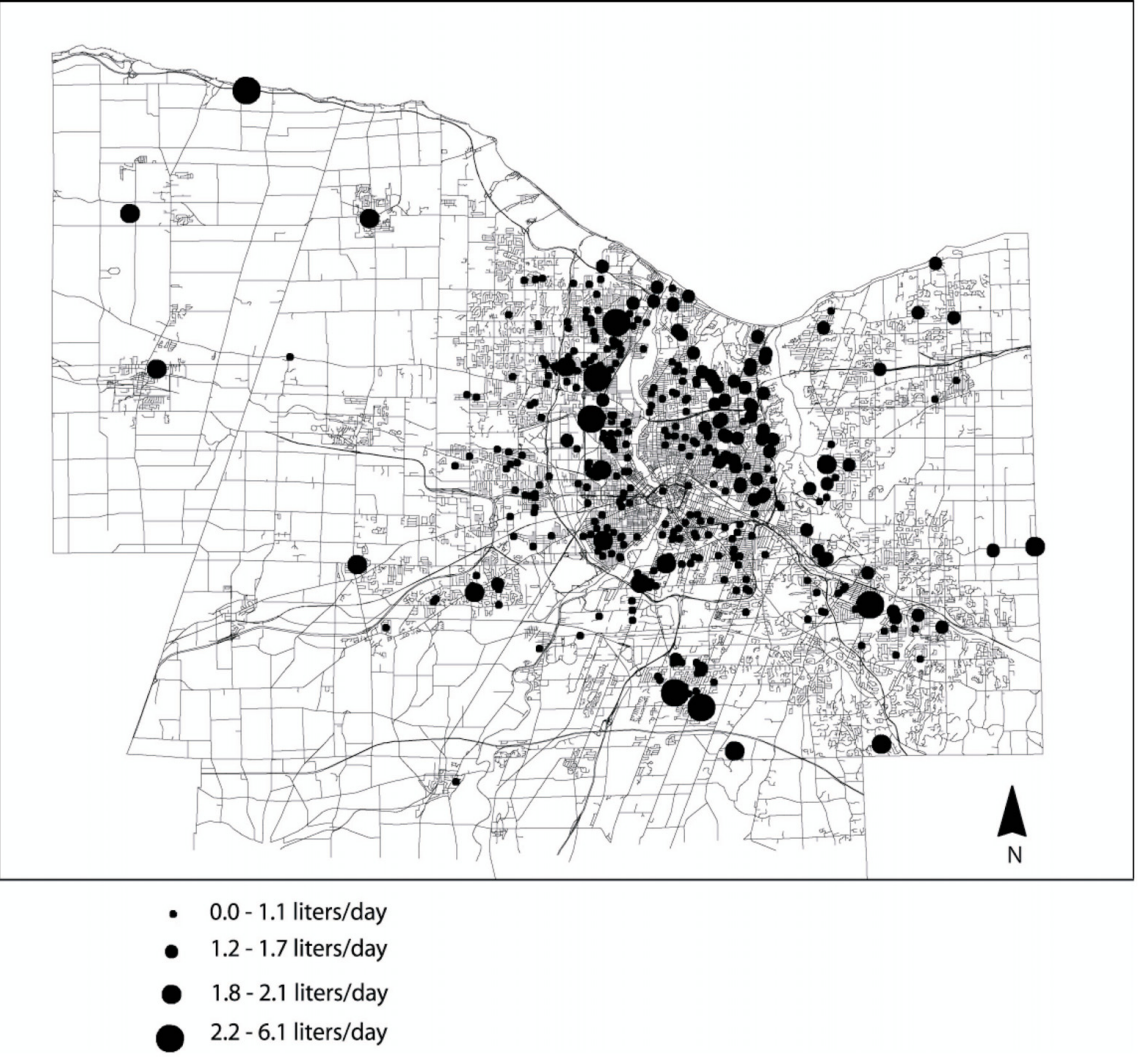
Average daily liters of tap water (ug/l) consumed by study participants.

Figure 3 shows the spatial variation in point estimates of rectal cancer risk, resulting from the bromoform model shown in Table 4. Geographic patterns of tap water consumption (one component of consumption exposure, which is estimated as a composite of bromoform concentrations and total tap water consumption) were not as marked (Figure 4) as the geographic distributions of estimated bromoform concentrations (Figure 1). Spatial trends of bromoform estimates closely corresponded to the geographic trend associated with OR's (Figure 3). This suggests that, although both are important, bromoform levels within the system likely had a greater effect on OR's than the amount of tap water that study participants consumed.

We also created quartiles based upon bromoform consumption estimates and found that after controlling for covariates, odds ratios were significantly increased in the fourth quartile of exposure to bromoform (OR 2.32, 95% CI : 1.22–4.39) (Table 5). The range of total daily consumption of bromoform for those in the highest quartile was quite wide (1.69 – 15.43 ug/l); however, upon closer examination, most individuals (seventy-five percent) consumed less than 5 ug/l per day, and only a small portion (seven percent) consumed greater than 10 ug/l per day.

**Table 5:** Quartiles of odds ratios^1 ^of risk for rectal cancer for total daily ingestion (ug/day) from tap water of bromoform from tap water in Monroe County, New York State

**Bromoform Quartile (ug/day)**	**OR (95%CI)**	**β**	***p *value**
**1 **(Low) (0.90–0.64)	-----	-----	-----
**2 **(0.65–0.97)	1.42 (0.73–2.74)	0.35	0.42
**3 **(0.98–1.68)	1.63 (0.85–2.69)	0.49	0.10
**4 **(High)(1.69–15.43)	2.32 (1.22–4.39)	0.84	0.01

1: abbreviations are as follows: OR, odds ratio; CI, confidence interval

2: exposure determined from consumption of tap water and estimated levels of bromoform (ug/l)

3: Results adjusted for covariates listed in Table 3

4: *p*-value for linear trend: 0.002

## Discussion

This is one of the first studies to use GIS in the exposure assessment to evaluate cancer risk associated with DBPs. The study uses a combination of individual tap water consumption data from a case control study, and geographic-based exposure analysis of disinfectant byproducts and finds increased risk for males exposed to the highest levels of bromoform. The dose-response pattern found for bromoform (Table 5) strengthens the association. Chlorodibromomethane and bromodichloromethane had elevated point estimates and confidence intervals that were marginally significant (OR = 1.78; 95% CI 1.00–3.19, and OR = 1.15; 95% CI 1.00–1.32, respectively).

Chlorinated drinking water contains a large range of byproducts produced during the disinfection process, and although many remain unidentified (accounting for more than fifty percent of the mass of total organic halides) [20], some have been established as carcinogenic. Of the brominated byproducts produced, bromodichloromethane, and bromoform have been shown to be carcinogenic in laboratory animals [45,46,53], and chlorine and chloramines were not [46]. Although colorectal cancer risk has been linked with a variety of ecological based water studies [27-29], with the exception of rodent studies, we found no strong previous evidence in the literature suggesting and/or supporting a specific association of rectal cancer risk with exposure to bromoform in humans. Studies of risk associated with exposure to DBPs are often based on indirect exposure assessments that rely on relatively large scales of geographic classifications of DBPs [54], and classification of exposed and unexposed groups is often made based on town, parish, or areas served by specific water treatment systems [28,29,31,42,55].

We attempted to refine the exposure assessments to improve upon previous studies, and to define a specific amount of DBP consumption that may lead to increased risk. By determining exposure as a mixture of water use patterns for individuals and a more specific estimate for DBPs at the tap, our study builds upon previous work [30,31,39] and attempts to examine risk from specific THM components and thereby avoid grouping all THMs together. In our refinement of the THM component of risk, bromoform, bromodichloromethane, and chlorodibromethane stood out as potentially important in the development of rectal cancer. Weaknesses in the approach we use to determine byproduct exposure estimates include (a) a time lag between case control data collection and DBP data collection, (b) the related concern that DBP data were not collected during the most relevant period of exposure, (c) consequences of residential mobility influencing exposure, and (c) kriging has been used on a network, despite being an interpolation method designed for planar data. Despite these limitations, some refinement of exposure in this hybrid study has been attained.

To assess the first two of these limitations, we plotted a time series of THM maps within GIS. From these plots we were able to determine that, at least during the time for which THM data were available (1998–2003), levels decreased slightly, and spatial distributions remained relatively unchanged [52]. In addition, Moran's Index calculated for THM sample sites indicated spatial clustering of values, with the greatest number of samples over 75 ug/l (for Total 551) occurring in the first half (1998–2000) of the sample period [52]. Moran's *I *did not change much over the period, indicating little change in the spatial pattern. In addition, Figure 2 indicates that, for the period 1986 to 2007, there is not a pronounced tendency for levels to change substantially over time (aside from seasonal variation). In addition, the lines in the figure, representing individual sample sites, do not cross one another in a clear and consistent manner, supporting the contention that spatial variation has not changed substantially.

Cancer studies of DBPs represent a unique challenge that often requires estimating past exposures without the support of relevant measurements [56]. Faced with this problem, many studies of drinking water and cancer base exposure estimates on geographic residence, partitioning space into areas that receive disinfected water and those that do not. Case control studies such as the one presented here, that attempt to determine the effects of exposures that likely take a considerable amount of time to produce an outcome, are subject to subtle or even large changes in the lifestyle of study participants (such as tap water consumption) that may occur over long time periods, and may not be readily recalled or accounted for. The WNY Diet Study data for cases and controls were collected over the period from 1979–1985. Mean age of study participants during the time of data collection was approximately 64 years. On average, potential exposure to DBPs started in the 1950's and continued until the time of interview. Our assumptions are similar in some respect to those in the original study that examined drinking water and cancer [49]. These assumptions are that water intake indicated during the case control interview is representative of water intake levels during a significant part of the participant's life span, and that spatial-based estimates of THM levels are reasonable representations of spatial patterns of THM distributions in the past. Regardless of how DBP exposure is determined, either by direct testing at the tap (essentially a 'snapshot' of DBP levels), or some attempt to model exposure based on historic and or current DBP levels [26,30] such assumptions regarding the spatial distributions of DBPs are required.

Residential mobility is unlikely to have had detrimental effects on our analyses. Cases and controls spent a large percentage of their lives at the water source indicated during interviews (Table 1). This reflects the relatively low mobility that characterizes the region.

Kriging DBP levels within a distribution system is a novel and potentially more accurate means of determining exposure in DBP cancer studies through spatial interpolation. Future work will be aimed at using network distance in place of planar distance in the spatial interpolation.

Although changes and reductions of some DBPs upon boiling are possible [57], we found no difference between hot water tap consumption and cold water tap consumption on rectal cancer risks in our study; this may be due in part to a sample size in the current study that may have been too small to measure such an effect. It may also be the case that bromoform is serving as a surrogate for exposure to some of the most mutagenic brominated forms of other DBPs such as MX that are not affected by volatization such as BMX-1, BMX-2, BMX-3 (58, 59).

Some studies [60,61] indicate that the causal relationship between rectal cancer and THMs remains an open question; however, "evidence is somewhat stronger for rectal cancer than colon cancer" [60]. Although many of the myriad possibilities of exposure routes, some of which have shown to significantly affect THM blood levels [62,63] (e.g. dishwashing, showering, swimming), have yet to be tested for their potential to influence risk, digestive tract cancers are more likely to be influenced by direct consumption than by inhalation or dermal routes. In addition, bromoform was not found to increase in the blood following certain daily activities [62].

Disinfectant byproducts in drinking water have garnered a considerable amount of attention since their discovery in 1975. Although many byproducts are thought to have carcinogenic potential, much of the focus has been on the trihalomethane group. Despite numerous clinical and field studies examining the effects on human health of DBP exposure, no conclusive paper has emerged that suggests what level of exposure might be acceptable. In the U.S.A, for many contaminants the Environmental Protection Agency establishes a health goal or maximum contaminant level (MCL). The goals are not necessarily legal limits with which water systems must comply. It should be noted that MCWA was well within compliance with federal standards for total trihalomethanes during the entire sampling period. At no time during the five-year testing period did the system exceed the previous (effective until 12/30/01) legal MCLs for total trihalomethanes of 0.10 mg/l^2^; the current standards for bromoform (0.08 ug/l^2^), were marginally exceeded only a handful of times for all samples.

It is certain that the present study has exposure misclassification, but we have no reason to believe that it is differential between cases and controls, and hence any misclassification should bias risk estimates towards the null. Nonetheless, we found elevated risk estimates for the highest estimated levels of exposure to THMs. Our study has the advantage of measures of individual intake of drinking water, and estimated exposure to THMs based on location of residence in the distribution system coupled with a study population with a stable residential history.

## Conclusion

A logistic regression model based on data for 128 cases of rectal cancer and 253 controls in Monroe County, NY, revealed increased risk for rectal cancer with increased levels of particular DBPs. In particular we found that locations with a high level of bromoform in the water distribution system corresponded with an increase in the odds ratio (OR = 1.85; 95% CI = 1.25 – 9.56) for rectal cancer. In addition to bromoform level at a given location, consumption of bromoform was also significant. The highest quartiles of estimated consumption of bromoform (1.69–15.43 ug/day) led to increased risk for rectal cancer (OR = 2.32; 95% CI = 1.22–4.39). Two other THMs were marginally associated with a possible increase in risk (chlorodibromomethane (OR = 1.78, 95% CI = 1.00–3.19) and bromodichloromethane (OR = 1.15; 95% CI = 1.00–1.32)).

## Competing interests

The author(s) declare that they have no competing interests.

## Authors' contributions

GB contributed to the conception of the study, and carried out the analyses and drafted the manuscript. PR contributed to the conception of the statistical analyses and participated in the design and coordination of the study. JV conceived the study and participated in its design and coordination. All authors read and approved the final manuscript.

## References

[B1] Parkin DM, Bray F, Farlay J, Pisani P (2005). Global Cancer Statistics, 2002. CA Cancer J Clin.

[B2] Devesa SS, Grauman DG, Blot WJ, Pennello G, Hoover RN, Fraumeni JF (1999). Atlas of Cancer Mortality in the United States, 1950–94.

[B3] Heineman EF, Zahm SH, Mclaughlin JK, Vaught JB (1994). Increased risk of colorectal-cancer among smokers-resluts of a 26-year follow-up of U.S. veterans and a review. Int J Cancer.

[B4] Chyou PH, Nomura AM, Stemmermann GN (1996). A prospective study of colon and rectal cancer amoung Hawaii Japanese men. Ann Epidemiol.

[B5] Freudenheim JL, Graham S, Marshall JR, Haughey BP, Wilkeson G (1990). A case-control study of diet and rectal cancer in Western New York. Am J Epidemiol.

[B6] Pedersen A, Johansen C, Gronbaek M (2003). Relations between amount and type of alcohol and colon and rectal cancer in a Danish population based cohort study. Gut.

[B7] Slattery ML, Sweeney C, Murtaugh M, Ma KN, Caan BJ, Potter JD, Wolff R (2006). Associations between vitamin D, vitamin D receptor gene and the androgen receptor gene with colon and rectal cancer. Int J Cancer.

[B8] Lynch HT, Lynch JF (1998). Genetics of colonic cancer. Digestion.

[B9] Dumas S, Parent ME, Siemiatycki J, Brisson J (2000). Rectal cancer and occupational risk factors: A hypothesis generating, exposure based case control study. Int J Cancer.

[B10] Weiderpass E, Vainio H, Kauppinen T, Vasama-Neuvonen K, Partanen T, Pukkala E (2003). Occupational exposures and gastrointestinal cancers among Finnish women. J Occup Environ Med.

[B11] Michels KB, Giovannucci E, Joshipura KJ, Rosner BA, Stampfer MJ, Fuchs CS, Colditz GA, Speizer FE, Willet WC (2000). Prospective study of fruit and vegetable consumption and incidence of colon and rectal cancers. J Natl Cancer Inst.

[B12] Matsu I, Yamamoto K, Harada H, Okada S (2002). Polymorphisms of NAT2 alcohol related enzyme encoding gene and rectal cancer. Jpn J Cancer Res.

[B13] Chao A, Thun MJ, Connell CJ, McCullough ML, Jacobs EJ, Flanders WD, Rodriguez C, Sinha R, Calle EE (2005). Meat consumption and risk of colorectal cancer. JAMA.

[B14] Fawell J (2000). Risk assessment case study-Chloroform and related substances. Food Chemical Toxicol.

[B15] Rook JJ (1975). Uproar about chlorine. Environ Sci Technol.

[B16] Rook JJ (1976). Haloforms in drinking-water. J Am Water Works Assoc.

[B17] Kuzma RJ, Kuzma CM, Buncher CR (1977). Ohio drinking water source and cancer rates. Am J Public Health.

[B18] Cantor KP, Hoover R, Manson TJ, McCabe K (1978). Association of cancer mortality with halomethanes in drinking water. J Natl Cancer Inst.

[B19] Boorman GA, Dellarco V, Dunnick JK, Chapin RE, Hunter S, Hauchman F, Gardner H, Cox M, Sills R (1999). Drinking water disinfection byproducts: review and approach to toxicity evaluation. Environ Health Perspect.

[B20] Simmons JE, Teuschier LK, Gennings C, Speth TF, Richardson SD, Miltner RJ, Narotsky MG, Schenck KD, Hunter ES, Hertzberg RC, Rice G (2004). Component-based and whole-mixture techniques for addressing the toxicity of drinking-water disinfection by-product mixtures. J Toxicol Environ Health A.

[B21] Clark RM, Thurnau RC, Sivaganesan M, Ringhand P (2001). Predicting the formation of chlorinated and bromated by-products. J Environ Eng.

[B22] Garciavillanova RJ, Garcia C, Gomez JA, Garcia MP, Ardanuy R (1997). Formation, evolution and modeling of trihalomethanes in the drinking water of a town in the distribution system. Water Res.

[B23] Hutton PH, Chung FI (1994). Correlating trihalomethane data. J Environ Eng.

[B24] Rossman LA, Brown BA, Singer PC, Nuckols JR (2001). DBP formation kinetics in a simulated distribution system. Water Res.

[B25] Bruemmer B, White E, Vaughan T, Cheney C (1997). Fluid intake and the incidence of urinary bladder cancer among middle-aged men and women in a three-county area of western Washington. Nutr Cancer.

[B26] King WD, Marrett LD (1996). Case-control study of urinary bladder cancer and chlorination by-products in treated water (Ontario, Canada). Cancer Causes Control.

[B27] Yang CY, Chiu HF, Cheng MF, Tsai SS (1998). Chlorination of drinking water and cancer mortality in Taiwan. Environ Res.

[B28] Chen K, Yu W, Ma X, Yao K, Jian Q (2005). The association between drinking water source and colorectal cancer incidence in Jiashan County of China: a prospective cohort study. Eur J Public Health.

[B29] Gottlieb MS, Carr JK, Morris DT (1981). Cancer and drinking water in Louisiana. A retrospective mortality study. Am J Epidemiol.

[B30] King WD, Marett LD, Woolcott CG (2000). Case-control study of colon and rectal cancers and chlorination by-products in treated water. Cancer Epidemiol, Biomarkers Prev.

[B31] Young TB, Wolf DA, Kannarek MS (1987). Case-control study of colon cancer and drinking water trihalomethanes in Wisconsin. Int J Epidemiol.

[B32] Koivusalo M, Jaakkola JJK, Vartiainen T, Hakulinen T, Karajalainen S, Pukkala E, Tuomisto J (1994). Drinking water mutagenicity and Gastrointestinal and urinary tract cancers: an ecological study in Finland. Am J Public Health.

[B33] Ahmed AE, Jacob S, Campbell GA, Harirah HM, Perez-Pollo JR, Johnson KM (2005). Fetal origin of adverse pregnancy outcome: The water disinfectant by-product chloroacetonitrile induces oxidative stress and apoptosis in mouse fetal brain. Developmental Brain Res.

[B34] Bove F, Shim Y, Zeitz P (2002). Drinking water contaminants and adverse pregnancy outcomes: A review. Environ Health Perspect.

[B35] Tardiff RG, Carson ML, Ginevan ME (2006). Updated weight of evidence for an association between adverse reproductive and developmental effects and exposure to disinfection by-products. Regul Toxicol Pharmacol.

[B36] Gottlieb MS, Carr JK, Clarkson JR (1982). Drinking water and cancer in Louisiana: A retrospective mortality study. Am J Epidemiol.

[B37] Bean JA, Isacson P, Hausler WJ, Kohler J (1982). Drinking water and cancer incidence in Iowa I. Trends and incidence by source of drinking water and size of municipality. Am J Epidemiol.

[B38] Brenniman GR, Vasilomanolakis-Lagos J, Amsel J, Namekata T, Wolff AH, Brungs WA, Cumming RB, Jacobs VA (1982). Case-Control study of cancer deaths in Illinois communities served by chlorinated or nonchlorinated water. Water chlorination: Environmental impact and health effects.

[B39] Hildesheim ME, Cantor KP, Lynch CF, Dosemeci M, Lubin J, Alavanja M, Craun G (1998). Drinking water source and chlorination byproducts II. Risk of colon and rectal cancers. Epidemiol.

[B40] Struba RJ Cancer and drinking water quality.

[B41] Savin JE, Cohn PD (1996). Comparison of bladder and rectal cancer incidence with trihalomethanes in driking water. Epidemiol.

[B42] Zierler S, Danley RA, Feingold L (1986). Type of disinfectant in drinking water and patterns of mortality in Massachusetts. Environ Health Perspect.

[B43] Morris RD, Audet AM, Angelillo IF, Chalmers TC, Mosteller F (1992). Chlorination, chlorination by-products, and cancer- a metaanalysis. Am J Public Health.

[B44] Carlo GL (1980). Organic drinking water contaminants and the incidence of selected gastrointestinal and urinary tract cancers. SUNYBuffalo (Thesis).

[B45] DeAngelo AB, Geter DR, Rosenberg DW, Crary CK, George MH (2002). The induction of aberrant crypt foci (ACF) in the colons of rats by trihalomethanes administered in the drinking water. Cancer Lett.

[B46] Dunnick JK, Melnick RL (1993). Assessment of the carcinogenic potential of chlorinated water – experimental studies of chlorine, chloramines, and trihalomethanes. J Natl Cancer Inst.

[B47] Shy CM (1985). Chemical contamination of water supplies. Environ Health Perspect.

[B48] Vena JE, Graham S, Freudenheim J, Marshall J, Zielezny M, Swanson M, Sufrin G (1993). Drinking water, fluid intake, and urinary bladder cancer in Western New York. Archives Environ Health.

[B49] Byers TE, Graham S, Haughey BP, Marshall JR, Swanson KS (1987). Diet and lung cancer risk: findings from the Western New York diet study. Am J Epidemiol.

[B50] Graham S, Haughey B, Marshall J, Brasure J, Zielezny M, Freudenheim J, West D, Nolan J, Wilkinson G (1990). Diet in the epidemiology of gastric cancer. Nutr Cancer.

[B51] (1990). U.S. Environmental Protection Agency. Methods for the Determination of Organic Compounds in Drinking Water, Supplement I. Environmental Monitoring Systems Laboratory. Office of Research and Development, Cincinnati, Ohio 45268 EPA-600/4-90/020.

[B52] Bove GE, Rogerson PA, Vena JE Case Control Study of the Effects of Trihalomethanes on the Risk for Urinary Bladder Cancer.

[B53] Komulainen H (2004). Experimental cancer studies of chlorinated by-products. Toxicology.

[B54] Wright MJ, Bateson TF (2005). A sensitivity analysis of bias in relative risk estimates due to disinfection by-product exposure misclassification. J Expo Anal Environ Epidemiol.

[B55] Kanarek MS, Young TB (1982). Drinking water treatment and risk of cancer death in Wisconsin. Environ Health Perspect.

[B56] Arbuckle TE, Hrudey SE, Krasner SW, Nuckols JR, Richardson SD, Singer P, Mendola P, Dodds L, Weisel C, Ashley DL, Froese KL, Pegram RA, Schultz IR, Reif J, Bachand AM, Benoit FM, Lynberg M, Poole C, Waller K (2002). Assessing Exposure in Epidemiologic Studies to Disinfection By-Products in Drinking Water: Report from an International Workshop. Environ Health Perspect.

[B57] Krasner SW, Mcguire MJ, Jacangelo JJ, Joseph G, Patania NL, Reagan KM, Kevin M, Aieta EM (1989). The occurance of disinfection by-products in U.S. drinking water. J Am Water Works Assoc.

[B58] McDonald TA, Komulainen H (2005). Carcinogenicity of the chlorination disinfection by-product MX. J Environ Sci Health Part C-Environ Carcinog Ecotoxicol Rev.

[B59] Suzuki N, Nakanishi J (1995). Brominated analogs of MX (3-Chloro-4-(Dichloromethyl)-5-Hydroxy-2(5H)-Furone) in chlorinated drinking water. Chemospehere.

[B60] Mills CJ, Bull RJ, Cantor KP, Reif J, Hrudey SE, Huston P, an expert working group (2000). Workshop report, health risk of drinking water chlorination by-products: report of an expert working group. Chronic Diseases in Canada.

[B61] Poole C (1997). Analytical meta-analysis of epidemiologic studies of chlorinated drinking water and cancer: quantitative review and re-analysis of the work published by Morris et al., Am J Public Health. Cincinnati, Ohio, US Environmental Protection Agency, National Center for Environmental Assessment.

[B62] Nuckols J, Lyu C, Hinckley A, Ashley D (2004). A study of the influence of household water quality and use activities on indoor air and internal dose levels of trihalomethanes – overview of methods and findings. Epidemiol.

[B63] Backer LC, Ashley DL, Bonin MA, Cardinali FL, Kieszak SM, Wooten JV (2000). Household exposures to drinking water disinfection by-products: whole blood trihalomethane levels. J Expo Anal Environ Epidemiol.

